# Mechanochromic Displays Based on Photoswitchable Cholesteric Liquid Crystal Elastomers

**DOI:** 10.1002/anie.202413559

**Published:** 2024-10-22

**Authors:** Lucas D. C. de Castro, Johan Lub, Osvaldo N. Oliveira, Albert P. H. J. Schenning

**Affiliations:** ^1^ São Carlos Institute of Physics University of São Paulo São Carlos SP 13560-970 Brazil; ^2^ Laboratory of Stimuli-responsive Functional Materials and Devices (SFD) Department of Chemical Engineering and Chemistry Eindhoven University of Technology Eindhoven The Netherlands; ^3^ Institute for Complex Molecular Systems Eindhoven University of Technology Eindhoven The Netherlands

**Keywords:** mechanochromic polymers, photoswitches, structural color, liquid crystal elastomers, cholesteric liquid crystals

## Abstract

Stimuli responsive optical materials are attractive for many areas, from healthcare to art design. However, creating intricate color‐changing patterns for visual information is still a challenge. This work describes the preparation of mechanochromic structural colored intricate pictures imprinted in cholesteric liquid crystal elastomers by using a chiral isosorbide molecular photoswitch. The photoswitch contains a photoisomerizable cinnamate moiety and was incorporated in a main chain liquid crystal oligomer with photopolymerizable acrylate end groups. After coating, the structural colored film was irradiated with ultraviolet (UV) light in air causing E/Z isomerization of the cinnamate units leading to a redshift of the structural color of the film. A grayscale photomask was used to spatially control the photoisomerization reaction and imprint colorful pictures such as portraits and landscapes, in the cholesteric liquid crystal films with high resolution. Photopolymerization in a nitrogen atmosphere led to a mechanochromic cholesteric liquid crystal elastomer with striking structural colors that blueshift upon strain. The sharp details of the patterns were preserved even under deformation and the system returned to the initial state upon strain removal. Our work offers a simple photoswitch approach to prepare stimuli responsive optical polymers imprinted with color‐changing pictures of unprecedented complexity.

## Graphical abstract

Mechanochromic intricate pictures with striking structural colors are imprinted in cholesteric liquid crystal elastomers by using a chiral isosorbide molecular photoswitch.

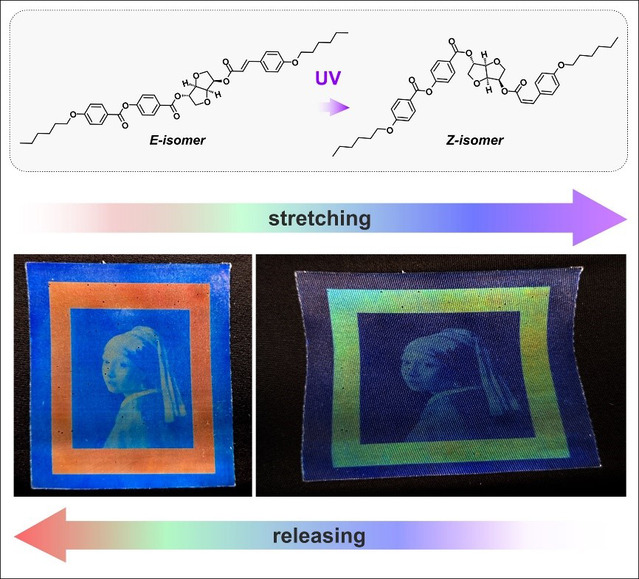

Stimuli responsive optical materials that undergo a programmed color change are employed in a variety of applications including actuators,[^1^, ^2^, ^3^, ^4^, ^5^, ^6^] smart textiles,[^7^, ^8^, ^9^, ^10^] sensors,[^11^, ^12^, ^13^] and biomedical devices.[^14^, ^15^] Cholesteric liquid crystal elastomers (CLCEs) are attractive among these optical materials because they combine the elastic behavior of rubbers with the programmable optical properties of cholesteric liquid crystals (CLCs). Striking structural colors arise from their self‐assembled helical structure induced by incorporation of a chiral dopant in nematic liquid crystals. The reflected wavelength depends on the concentration and helical twisting power (HTP) of the chiral dopant. For creating colorful patterns for visual information, one may exploit a photomask and crosslinking areas of the sample at different temperatures but this approach is laborious.[^16^, ^17^, ^18^, ^19^] On the other hand, alternative strategies to prepare multicolored mechanochromic films lack resolution (Table S1). Therefore, producing high quality optical structures in stimuli responsive optical materials remains challenging.

Light as a trigger can induce a color change in photopolymers with high spatiotemporal precision.^[20]^ This is done with photoswitches, i.e. organic molecules that can be switched between two isomers when irradiated with suitable wavelengths.[^21^, ^22^] The molecular shape changes induced by photoisomerization can be amplified into macroscopic color change.[^23^, ^24^] For instance, static cholesteric layers that reflect different colors in different areas can be prepared by using chiral dopants with photoisomerizable double bonds, such as azobenzene, menthone^[25]^ and cinnamate derivatives.[^26^, ^27^] For most photoisomerizable chiral compounds, the E and the Z isomers have different HTPs and under ultraviolet (UV) light irradiation, isomerization takes place which affects the pitch of the CLC mixture.

Although molecular photoswitches have been used in static CLC films,[^28^, ^29^, ^30^] to the best of our knowledge they are rarely reported in stimuli responsive optical polymer materials. Herein, we report a rapid, straightforward strategy to imprint full‐colored mechanochromic pictures in CLCEs by using a photoswitchable molecule. Mechanochromic polymers are a class of stimuli responsive optical materials that change color in response to a mechanical stimulus.[^31^, ^32^]

To prepare the mechanochromic polymer, an acrylate‐terminated CLC oligomer containing a cinnamate derived chiral dopant photoswitch was synthesized (Figure 1). The reflection wavelength of the CLC oligomer was adjusted to be in the blue color wavelength region by controlling the amount of chiral dopant. The degree of polymerization (DP) of around 2.1 was calculated from the ^1^H‐nuclear magnetic resonance (^1^H NMR) spectrum (Figure S1) and a cholesteric‐to‐isotropic transition temperature (T_Ch−I_) of 88 °C was determined from the differential scanning calorimetry (DSC) thermogram (Figure S2). To prepare the ink for making the colorful patterns, the oligomer, a photoinitiator and chain transfer agent to tune the crosslinking degree^[33]^ were dissolved in dichloromethane (DCM) (Figure 1). The ink was deposited via bar coating on a polyethylene terephthalate substrate coated with poly(vinyl alcohol) (PET/PVA) using a procedure earlier described.^[34]^ A shear‐induced planar alignment was achieved during bar‐coating and a blue‐reflecting CLC layer was immediately obtained after the solvent evaporation.

**Figure 1:**
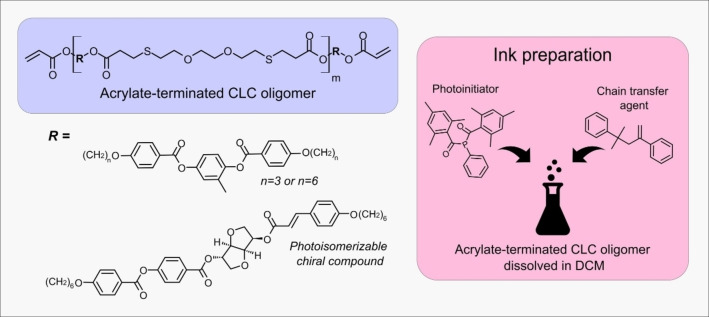
Chemical structure of the acrylate terminated CLC oligomer containing a cinnamate chiral dopant photoswitch (left) and a schematic illustration of the ink preparation, which includes a DCM solution containing a photoinitiator, a chain transfer agent and the acrylate terminated CLC oligomer (right).

To study the photo‐isomerization, the blue‐reflecting CLC film was irradiated with UV light with a dose of 180 mJ cm^−2^ in an air atmosphere (Figure 2a) resulting in an iridescent red color (Figure 2b). When irradiated with UV light, the photoswitchable cinnamate chiral compound might undergo a E/Z isomerization reaction (Figure 2c), where the isomerization is proportional to the dose.^[27]^ The inhibiting effect of oxygen on the radical polymerization of acrylates avoids the oligomer crosslinking. Therefore, the mobility of the oligomer might be maintained, and a rapid change of the cholesteric structure was enabled by the isomerization of the chiral compound. To confirm this hypothesis ^1^H‐nuclear magnetic resonance (^1^H NMR ) spectra of the oligomers before and after irradiation (Figure S1) were measured. Indeed, the acrylate end groups of the oligomers remain unaffected after irradiation. The doublet peaks at 7.3 and 7.47 ppm, which refers to the signal of the protons H^A^ and H^E^ on the aromatic rings, exhibit a 1 : 1 ratio of the integral values, indicating the presence of 100 % of the E‐isomer before UV irradiation (Figure 2d). After UV irradiation, the relative amount of H^E^ is reduced, since this proton will give a signal at 7.73 ppm (H^Z^ in Figure 2c) in the case of the Z‐isomer, while the signal of H^A^ is not affected by the isomerization reaction.^[27]^ This confirms that photoisomerization has partially occurred, leading to a mixture of E and Z isomers and the conversion degree ($C_{E/Z}$ ) calculated from the ^1^H NMR spectra was around 31 %. The reflection wavelength $\left(\lambda \right)$ of the red reflective film can be defined as:

(1) $$\lambda =n\;p=n{\left({HTP}_{E}x_{E}+{HTP}_{Z}x_{Z}\right)}^{-1}\;$$

**Figure 2:**
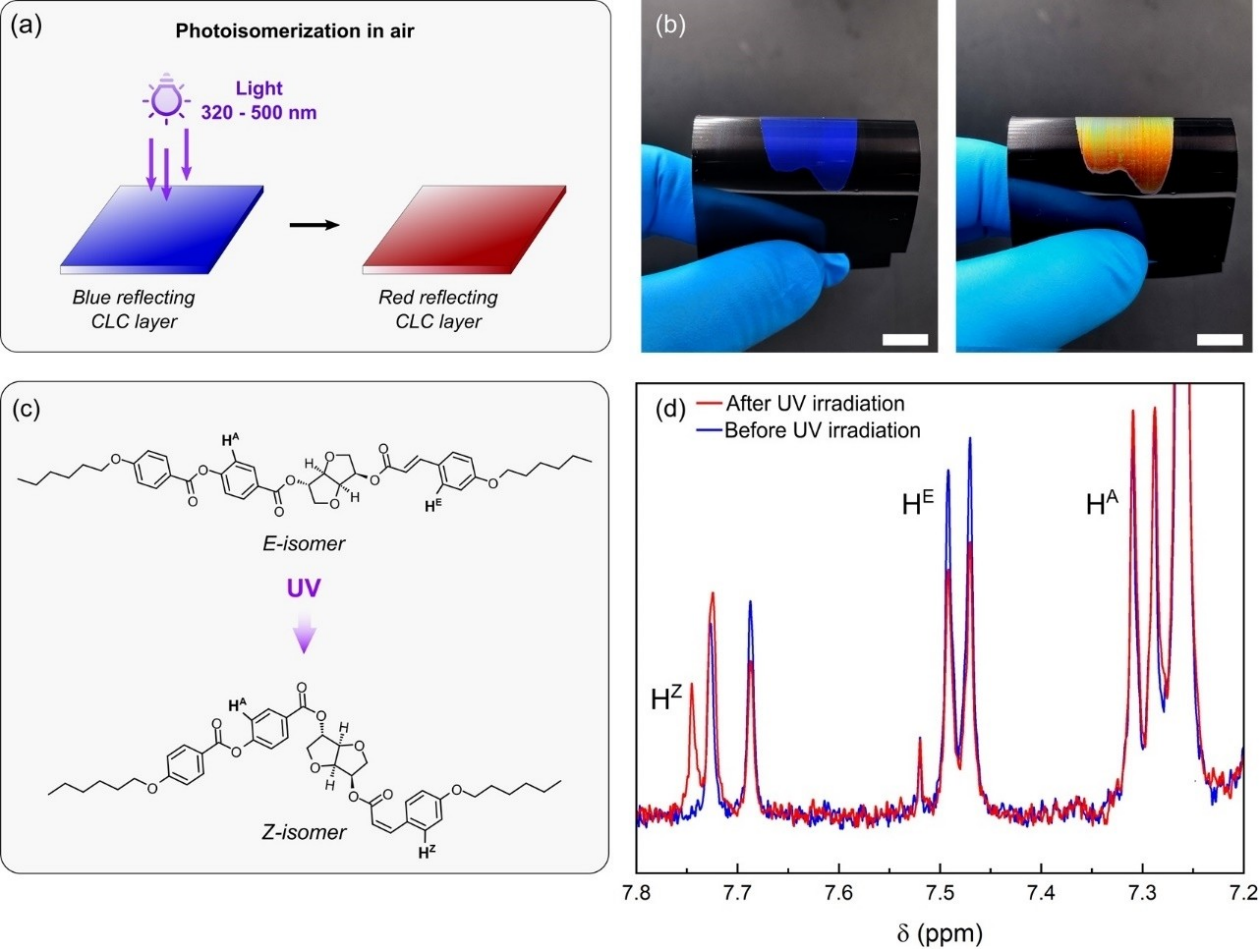
Color changes of the CLC ink before photopolymerization in a nitrogen atmosphere. (a) Schematic illustration of the photoisomerization step conducted in air atmosphere using UV light. (b) Digital pictures of the CLC layer before (left) and after (right) the UV irradiation. Scale bars are 1 cm. (c) Schematic illustration of the E/Z isomerization reaction of the cinnamate‐containing moiety in the oligomer when irradiated with UV light and position of the protons H^A^, H^E^ and H^Z^. (d) Excerpt from the ^1^H NMR spectra of the CLC layers before and after UV irradiation.

where n is the average refractive index of the cholesteric mixture; p is the pitch of the helix of the cholesteric structure; $x_{E}$ and $x_{Z}$ are respectively the weight fraction of E and Z isomers after the photoisomerization step; ${HTP}_{E}$ is the helical twisting power of the E‐isomer, defined as the reciprocal of p when $x_{E}=1$ ; and ${HTP}_{Z}$ is the helical twisting power of the Z‐isomer.^[27]^ Since the helical twisting power of the Z‐isomer is low,^[26]^ it seems reasonable to assume ${HTP}_{Z}$ =0 as an estimation. Before the photoisomerization step, the blue‐reflecting CLC layer exhibited a λ =445 nm. Thus, a $C_{E/Z}$ =31 % would result in a theoretical redshift to λ =645 nm, which is very close to the value of λ =638 nm experimentally determined by UV/Vis reflectance measurements (Figure S3).

Subsequently, the photopolymerization step was performed by irradiation with UV light in a nitrogen (N_2_) atmosphere. To avoid further color shifting, the process was carried out using a cut‐off filter of 400 nm in between the sample and light source (Figure 3a). The low absorbance of the chiral compound in the visible region almost inhibits E/Z isomerization^[26]^ and the photopolymerization occurred without significant changes in the helical structure (Figure S4). The photopolymerization conversion was confirmed with Fourier‐transform infrared spectroscopy (FTIR) using the C−H out‐of‐plane vibration of the acrylate group at 812 cm^−1^. To prevent an inelastic behavior,^[33]^ the incorporation of the chain transfer agent was applied (Figure 1) to control the crosslinking degree (Figure S5). For our system, the reduction of the crosslinking degree promoted by incorporation of 1 wt % chain transfer agent enabled a stable elastomeric behavior, and a CLCE with mechanochromic properties was obtained. The modulus CLCE is estimated to be in the order 100–800 MPa, depending upon the crosslinking degree.^[35]^ After being transferred to a stretchable substrate, the CLCE reflects a strong red color that blueshifts upon strain in a reversible manner (Figure 3b). The mechanochromic response is similar to earlier reported systems.[^34^, ^36^, ^37^] When the CLCE is stretched to 40 %, the reflection wavelength shifted from 638 nm to 551 nm and the relative color spectral shift ($\Delta \lambda /\lambda_{0}$ ) was 14 % (Figure S6a and Figure S6c). Moreover, the spectral shift is maintained even during sequential stretching and releasing cycles (Figure S6b).

**Figure 3:**
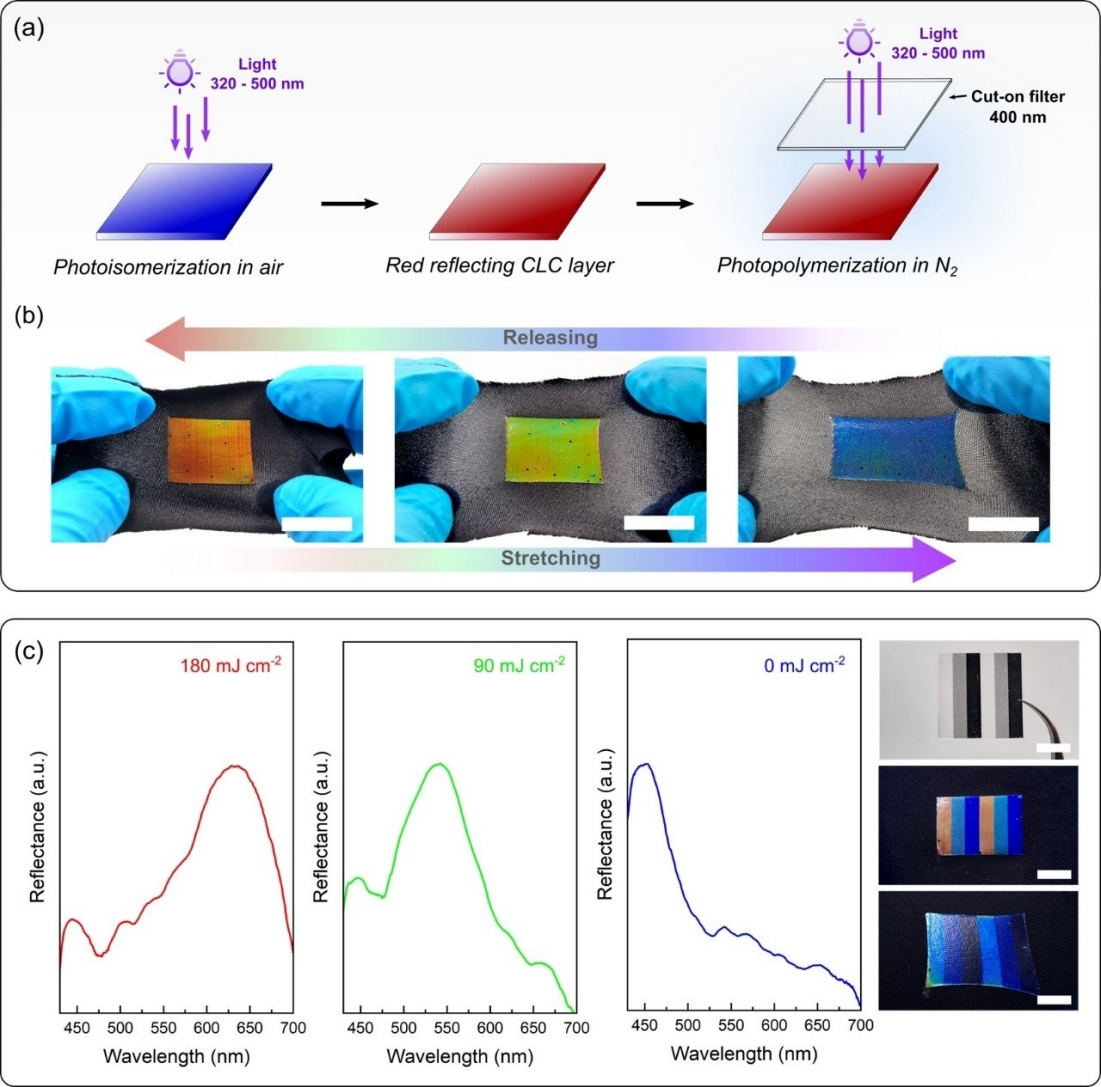
Mechanochromic behavior of the CLCE after photoisomerization and photopolymerization. (a) Schematic illustration of the photoisomerization in air and photopolymerization step conducted in N_2_ atmosphere. A cut‐off filter of 400 nm is placed in between the sample and light source to avoid further photoisomerization during photopolymerization. (b) Digital pictures of the red CLCE attached to a stretchable substrate. Scale bars are 2 cm. (c) Mechanochromic RGB pattern. A grayscale mask containing stripes with 100 %, 50 % and 0 % transmittance was placed in between the sample and light source during the photoisomerization step to control the UV dose and prepare red, green, and blue colors, respectively, as confirmed by the reflectance spectra and digital pictures. After photopolymerization, the CLCE imprinted with the RGB pattern exhibited mechanochromic behavior. Scale bars are 1 cm.

Since the conversion of E‐isomer to Z‐isomer is proportional to the irradiated UV dose and the relative concentration between both isomers determines the reflection wavelength, colorful patterns can be prepared in a single step through the control of UV intensity by a photomask during photoisomerization. For instance, a grayscale mask containing stripes with 100 %, 50 % and 0 % transmittance can imprint respectively the red, green, and blue colors in a blue reflecting CLC layer. As described above, the required UV dose to obtain the red color was around 180 mJ cm^−2^ and to obtain the green about half of that (Figure 3c and Figure S7). After crosslinking, the RGB stripes exhibit reversible mechanochromic behavior and the colors change when the CLCE is stretched (Figure 3c and Movie S1). The color tones are visible in every external lighting conditions. In this study, the digital pictures and videos were acquired using an ordinary tabletop light box equipped with white LEDs. The mechanochromic response of the different structural colors is the similar as observed for the red colored film (Figure 3b).

The spatiotemporal precision of the light trigger can be further explored to imprint mechanochromic pictures with remarkable resolution in the order of a few micrometers (Figure S8). The reflection wavelengths are dictated by the irradiated UV dose, which can be controlled using photomasks prepared on a regular laser printer. For instance, using a customized photomask we imprinted the painting entitled *Girl with a Pearl Earring*, from the Dutch artist Johannes Vermeer. After photoisomerization, the molecular orientation was further enhanced by an annealing step at 70 °C for 10 s. A very short annealing step below the T_Ch−I_ increases the molecular mobility and accelerates the relaxation of domain walls in the CLC, which reduces the number of orientation defects in the cholesteric layer.^[38]^ In turn, heating above the T_Ch−I_ destroys the cholesteric structure while longer annealing steps results in dewetting of the oligomer on the PET substrate and increases the number of defects on the CLC layer without a significant enhancement in the saturation of the reflected colors. A final photopolymerization step in N_2_ atmosphere enables a reversible mechanochromic behavior (Figure 4a). The picture features sharp details with striking structural colors that include red, different tones of light green and blue. The patterned CLCE has mechanochromic behavior and preserves the details without resolution deterioration even when stretched (Figure 4b and Movie S2). As expected, the apparent colors depend upon the applied strain and can be either in the visible range or in the UV region, which is invisible to the naked eye. With the aid of customized photomasks, the CLCEs can be prepared with a variety of intricate patterns. To further illustrate the versatility of our strategy, we also imprinted a picture of Amsterdam landscape (Figure 4c and Movie S3). The picture has structural colors with several tones of red, orange, yellow, green and blue that blueshift upon strain. Moreover, the mechanochromic behavior is fully reversible and the system returns to the initial state immediately after the strain is released.

**Figure 4:**
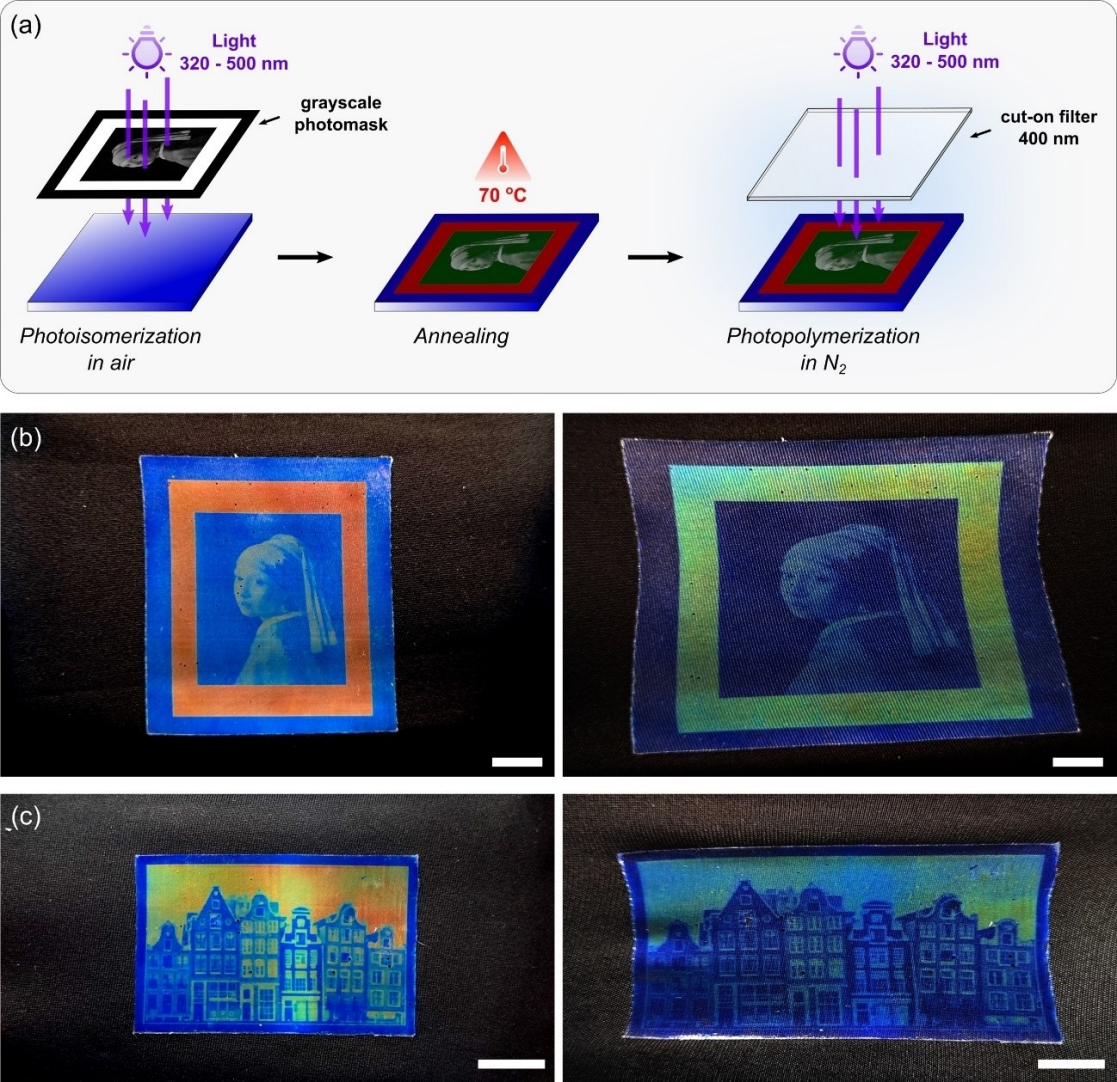
Mechanochromic pictures imprinted on CLCEs. (a) Strategy for imprinting pictures on a CLC layer and subsequent photopolymerization step. (b) Mechanochromic behavior of the CLCE imprinted with the portrait of the *Girl with a Pearl Earring*. When the patterned CLCE is stretched, the structural colors blueshift and the picture preserves the sharp details even under deformation. (c) Mechanochromic behavior of the CLCE imprinted with a picture of Amsterdam landscape. Scale bars are 1 cm.

In conclusion, we have demonstrated a straightforward strategy to prepare mechanochromic images imprinted on cholesteric liquid crystal elastomers by using a photoisomerizable isosorbide derivative as the reactive chiral dopant. Upon UV irradiation, the photoswitchable molecule undergoes an E/Z isomerization reaction in which the conversion from E‐isomer to Z‐isomer is proportional to the UV dose. Since the HTPs of the two isomers are different, the reflection wavelength of the CLC ink is dictated by their relative concentration. Through the spatial control of the E/Z conversion degree, one may use photomasks during photoisomerization to generate customized pictures imprinted on CLC layers in a single step. Although boundary defects related to the UV light scattering caused by the photomask during the photoisomerization step might appear, they are imperceptible to the naked eye and the intricate patterns exhibit remarkable resolution in the order of few microns. Because of the low absorbance of the chiral compound at higher wavelengths, a cut‐on filter of 400 nm enables a photopolymerization step without noticeable color changing. Photopolymerization renders the patterned CLCE a reversible mechanochromic behavior with striking structural colors that blueshift when stretched. The sharp details of the pictures are preserved even under deformation and the system returns to the initial state immediately after the strain is released. Our system points towards a new photoswitch method to make stimuli responsive optical materials with colorful patterns for visible information that may find application in sensors, smart textiles, security labels, or even in art design.

## Conflict of Interests

The authors declare no conflict of interest.

## Supporting information

As a service to our authors and readers, this journal provides supporting information supplied by the authors. Such materials are peer reviewed and may be re‐organized for online delivery, but are not copy‐edited or typeset. Technical support issues arising from supporting information (other than missing files) should be addressed to the authors.

Supporting Information

Supporting Information

Supporting Information

Supporting Information

## Data Availability

The data that support the findings of this study are available from the corresponding author upon reasonable request.
