# Supplementary material for: Mechanochromic Displays Based on Photoswitchable Cholesteric Liquid Crystal Elastomers
Source: Angew Chem Int Ed Engl. 2024 Oct 22;64(1):e202413559. doi: 10.1002/anie.202413559 (PMC11701355; doi:10.1002/anie.202413559)
Supplement: Supplementary file 1 — Supporting Information [file ANIE-64-e202413559-s001.pdf]

Angewandte Chemie
Eine Zeitschrift der Gesellschaft Deutscher Chemiker

Supporting Information

Mechanochromic Displays Based on Photoswitchable Cholesteric Liquid Crystal Elastomers

L. D. C. de Castro, J. Lub, O. N. Oliveira Jr, A. P. H. J. Schenning**

SUPPORTING INFORMATION

MECHANOCHROMIC DISPLAYS BASED ON PHOTOSWITCHABLE CHOLESTERIC LIQUID CRYSTAL ELASTOMERS

Lucas D. C. de Castro[1,2], Johan Lub[2], Osvaldo N. Oliveira Jr*[,1], and Albert P. H. J. Schenning[*,2,3]

[1]São Carlos Institute of Physics, University of São Paulo, São Carlos, SP, Brazil

[2]Laboratory of Stimuli-responsive Functional Materials and Devices (SFD), Department of Chemical Engineering and Chemistry, Eindhoven University of Technology, Eindhoven, The Netherlands

[3]Institute for Complex Molecular Systems, Eindhoven University of Technology, Eindhoven, The Netherlands

Corresponding authors: chu@ifsc.usp.br and a.p.h.j.schenning@tue.nl

EXPERIMENTAL SECTION

Materials: 2-methyl-1,4-phenylene bis(4-((6-(acryloyloxy)hexyl)oxy)benzoate) (RM82); and 2-methyl-1,4-phenylene bis(4-(3-(acryloyloxy)propoxy)benzoate) (RM257) were purchased from Daken Chemical. The isosorbide derivatives chiral compound (3S,3aR,6R,6aR)-3-(4-(4-(6-Acryloyloxyhexyloxy)benzoyloxy)benzoyloxy)hexahydro-furo[3,2-b]furan-6-(4-(6-acryloyloxyhexyloxy)cinnamate) (Photoisomerizable chiral compound) was synthesized according to an earlier reported procedure. [1] 2,2′-(Ethylenedioxy)diethanethiol (EDDET); dipropylamine (DPA); 2,4-Diphenyl-4-methyl-1-pentene (α-MSD) and polyvinyl alcohol (PVA, M_w 31,000 - 50,000) were purchased from Sigma-Aldrich. Irgacure 819 was purchased from Ciba Specialty Chemicals. Dichloromethane (DCM) was purchased from Bio Solve.

Preparation of the colorful CLCEs: In a typical procedure, 700 mg RM82, 200 mg RM257, 82 mg photoisomerizable chiral compound, 126 mg EDDT, and 10 µL of DPA were mixed in 5 mL DCM and stirred overnight at room temperature. The oligomer solution was dried overnight in a vacuum oven at 60°C. Prior to use, the ink was prepared with a mixture of 32 wt% dry oligomer, 66 wt% DCM, 1 wt% α-MSD, and 1 wt% Irgacure 819. A PVA layer (10 wt% in H_2O) was deposited with a bar coater (RK K control coater, 4 µm wire bar) on a PET foil and dried for 20 min at 70°C. Subsequently, the ink was deposited on the PET/PVA foil (12 µm wire bar) and dried for 30 min at room temperature to obtain a blue-reflecting cholesteric layer. Photoisomerization was performed by irradiation of ultraviolet (UV) light (OmniCure S2000 UV Curing System) for 10 min with an intensity of 0.3 mW cm^{-2} under air atmosphere, totalizing a 180 mJ cm^{-2} dose. This procedure was optimized to promote a color change from blue to red and performed in all photoisomerization reactions unless noted otherwise. The UV dose was spatially controlled by grayscale photomasks and explored to imprint colorful patterns. After the photoisomerization reaction, the molecular orientation can be further enhanced by a 10 s annealing step at 70°C. Crosslinking was performed by irradiation of UV light for 10 min with an intensity of 15 mW cm^{-2} under nitrogen (N_2) atmosphere. To avoid further color changing, a cut-on 400 nm filter (FSQ-GG400, Newport) was placed in-between the sample and light source during the crosslinking step. The mechanochromic prototypes were prepared by transferring the CLCEs to stretchable substrates, as described before. [2]

Characterization: ^{1}H-nuclear magnetic resonance (^{1}H-NMR) analysis was performed on a Bruker Avance Core III 400 MHz spectrometer using deuterated chloroform as the solvent. Fourier-transform infrared spectroscopy (FTIR) measurements were performed in a Varian 670 FT-IR spectrometer with a slide-on ATR (Ge). Differential scanning calorimetry (DSC) was carried out with a TA Instruments Q2000 operated a scan rating of 10°C min^{-1}. Optical micrographs were taken with a Leica DM2700 M polarized optical microscope (POM). Reflectance spectra were acquired with an Ocean Optics HR2000+ fiber optic spectrometer equipped in the POM.

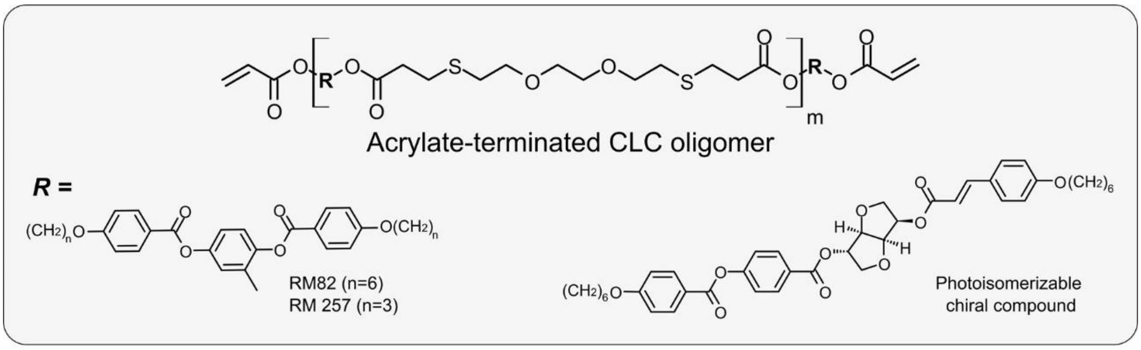

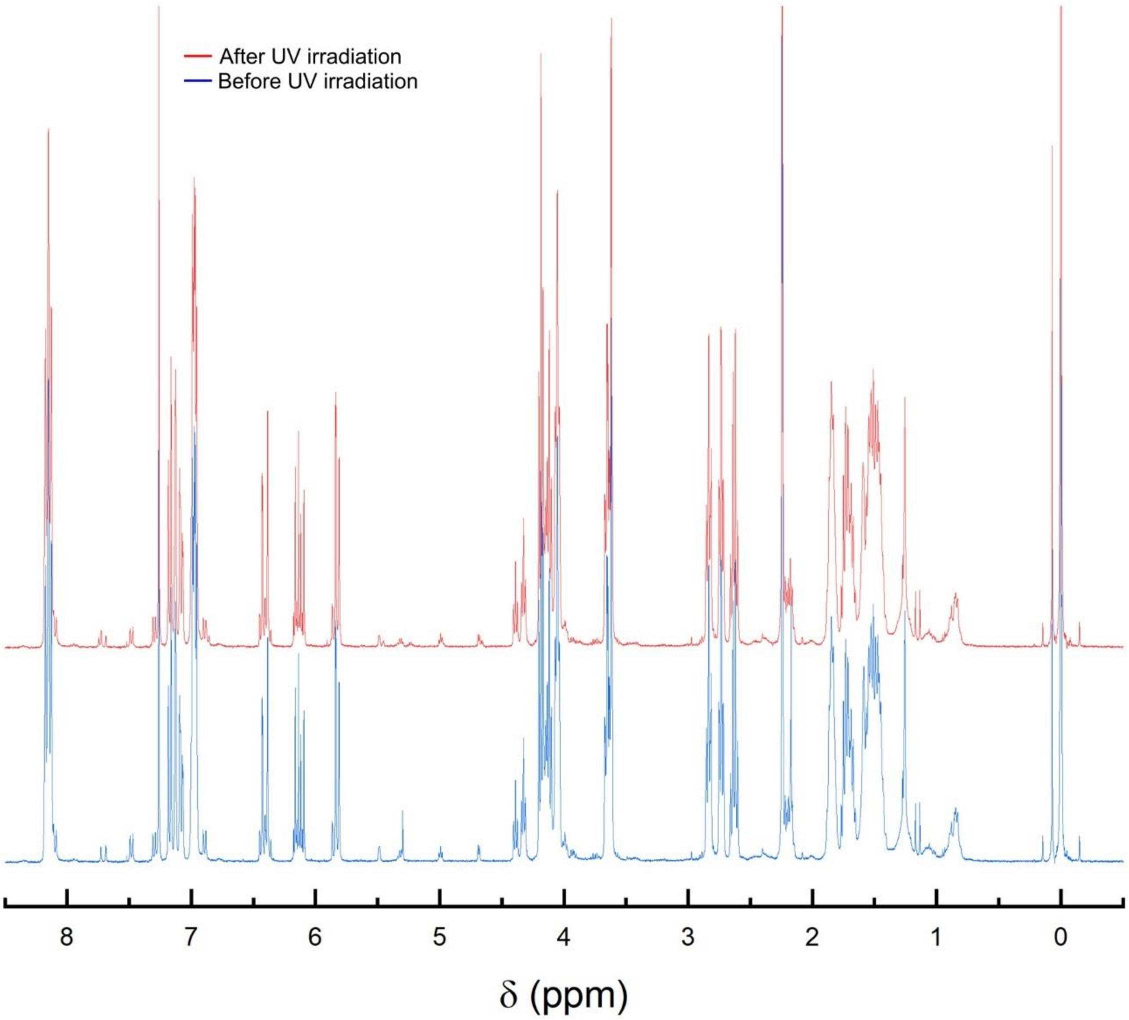

Fig. S1 ^{1}H-nuclear magnetic resonance (^{1}H-NMR) spectra of the acrylate CLC oligomers before and after UV irradiation with 180 mJ cm^{-2} in air atmosphere. The conversion degree $(C_{E/Z})$ can be defined as:

$$C_{E/Z} = x_E/(x_E + x_Z) \qquad \text{(S1)}$$

where x_E and x_Z are the weight fraction of E and Z isomers, respectively, after the photoisomerization step.

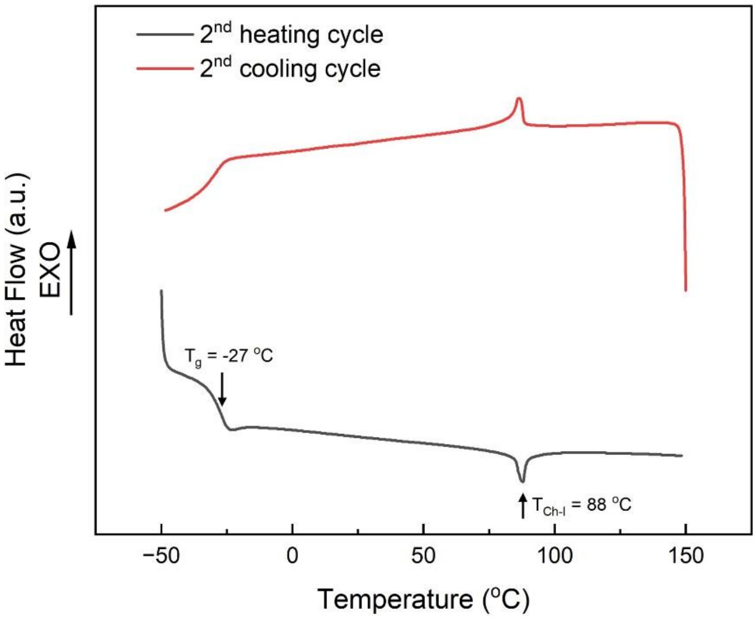

Fig. S2 Differential scanning calorimetry (DSC) thermogram of the CLC oligomer (second heating-cooling cycle). The glass transition temperature (T_g) is -27 ^oC and the cholesteric to isotropic transition temperature ($T_{Ch\text{-}I}$) is 88 ^oC.

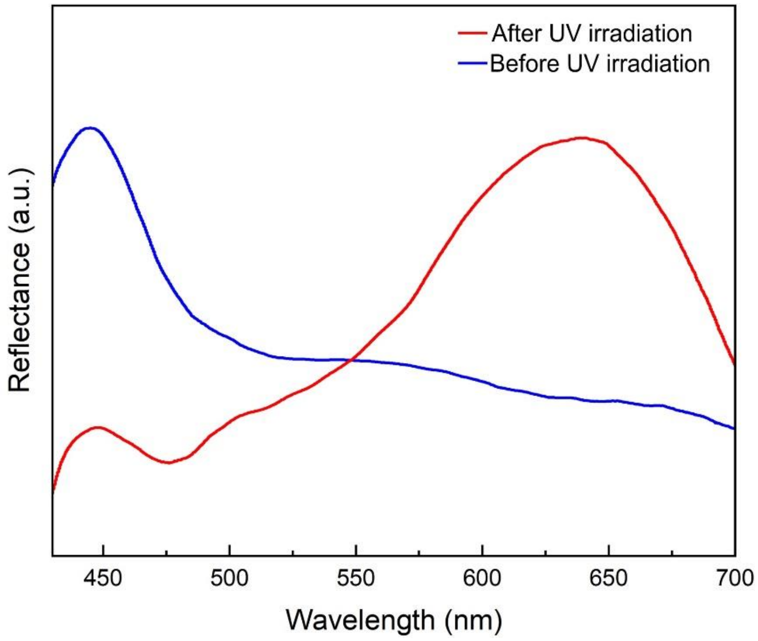

Fig. S3 UV-vis reflectance spectra of the acrylate CLC oligomer film before and after UV isomerization. When the blue-reflecting CLC layer is irradiated with 180 mJ cm^{-2}, a redshift of the reflection wavelength from 445 nm to 638 nm was observed.

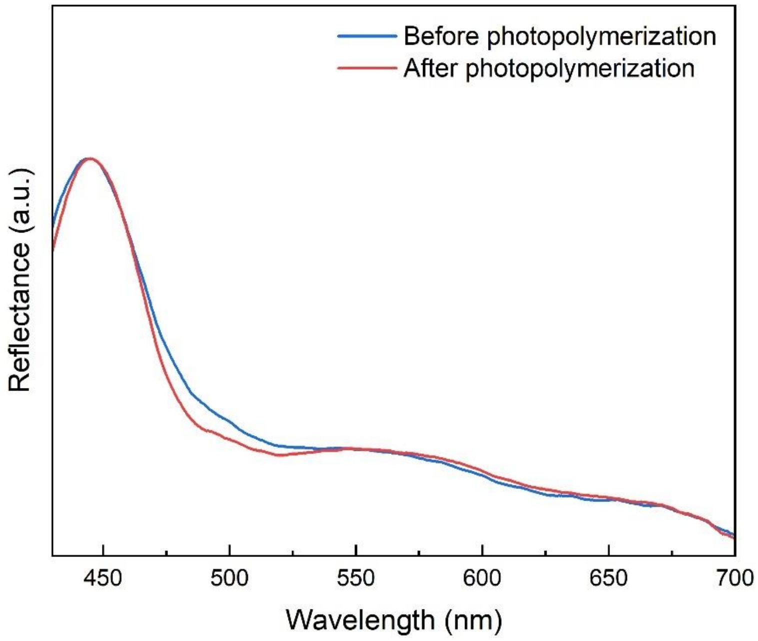

Fig. S4 Reflectance spectra of a blue reflecting CLC layer before and after photopolymerization carried out using a cut-off filter of 400 nm in between the sample and UV light source. No significant changes in the reflected colors were observed.

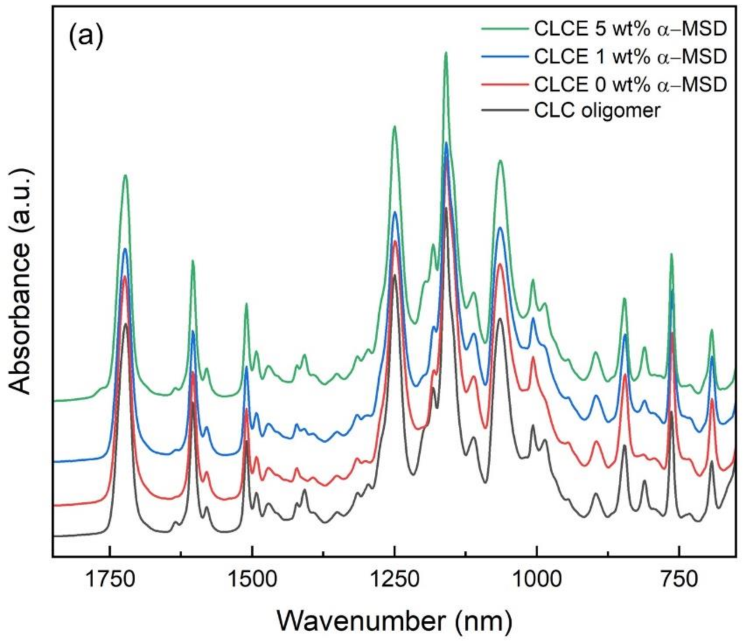

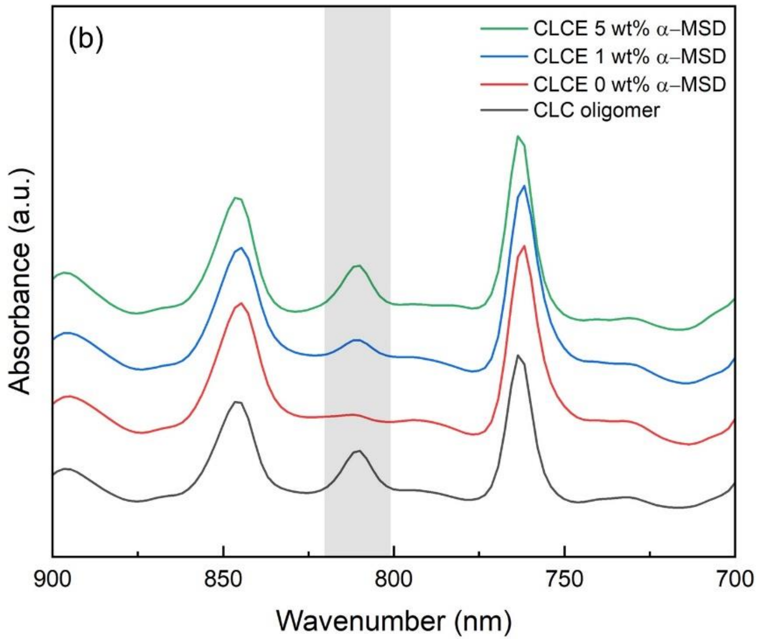

Fig. S5 (a) Full and (b) zoomed-in FTIR spectra of the CLCE containing different α-MSD concentrations. The CLC oligomer spectrum is also plotted as reference. Photopolymerization can be monitored using the C-H out-of-plane vibration of the acrylate group at 812 cm^{-1} highlighted as a shaded area in (b). The crosslinking degree is inversely proportional to the concentration of chain transfer agent. For our system, the incorporation of 1 wt% α-MSD enabled a stable elastomeric behavior while the incorporation of 5 wt% α-MSD completely inhibited photopolymerization.

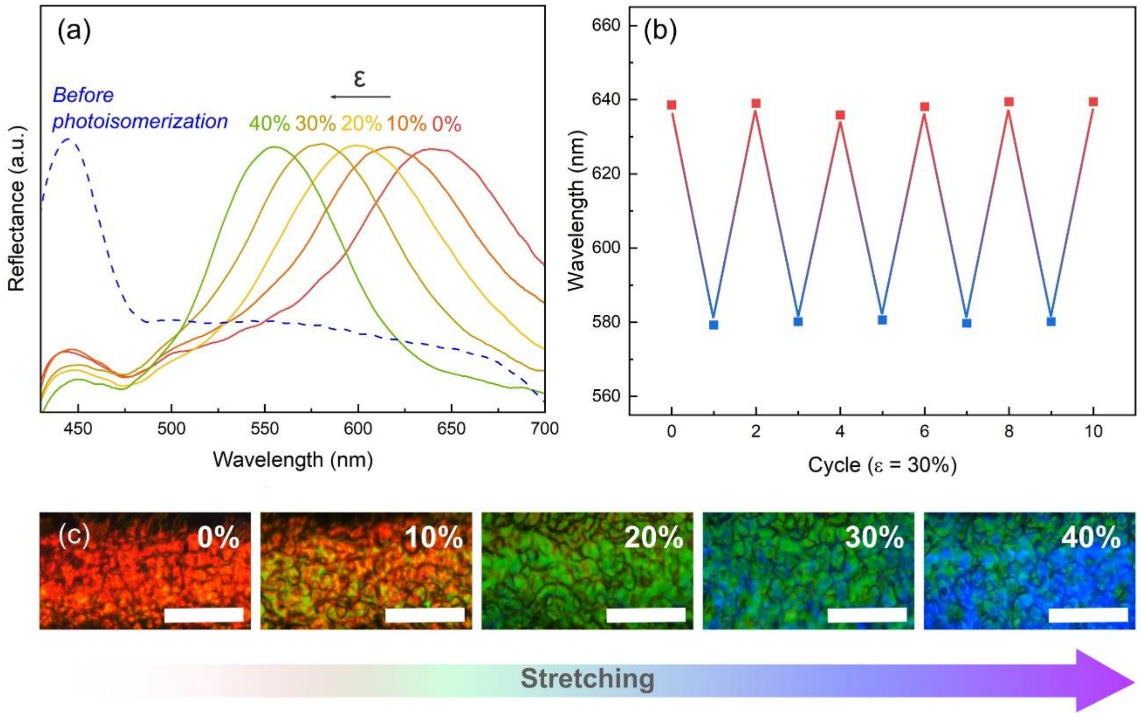

Fig. S6 Mechanochromic behavior of the CLCE prepared after photoisomerization with 180 mJ cm^{-2} and after photopolymerization. (a) Reflectance spectra recorded under uniaxial strain. For reference, the reflectance curve of the CLC ink before the photoisomerization step is plotted in dashed blue. (b) Reversible color-changing of the CLCE submitted to stretching-releasing cycles (ε = 30%). (c) POM images of the CLCE submitted to uniaxial strain. Scale bars are 25 μm.

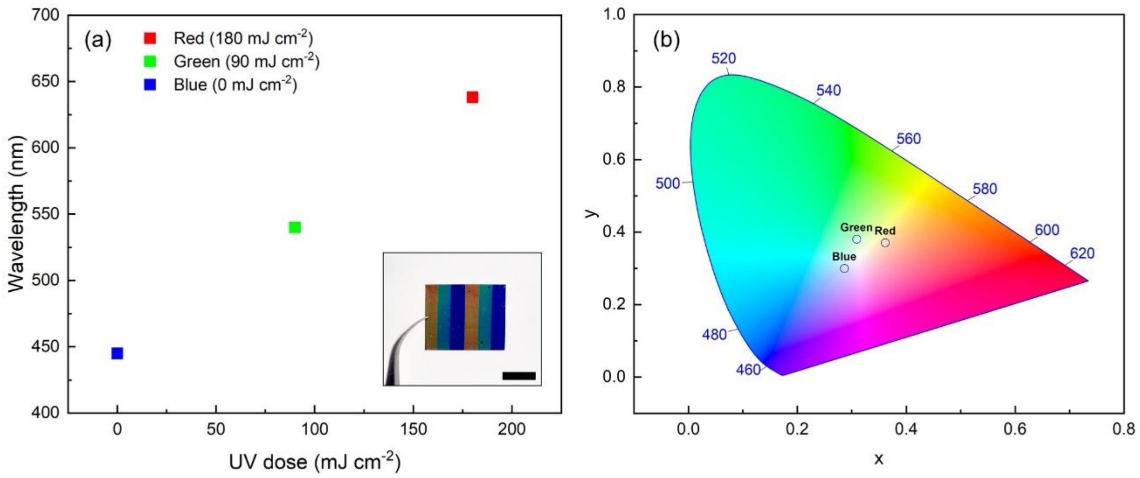

Fig. S7 (a) Reflection wavelengths of the red, green and blue colors as a function of the irradiated UV dose. λ values were obtained from the reflectance spectra on Fig. 3c. The inset shows a picture of a CLC layer imprinted with the RGB pattern. Scale bar is 1 cm. (b) CIE chromaticity diagram indicating the corresponding red, green and blue colors.

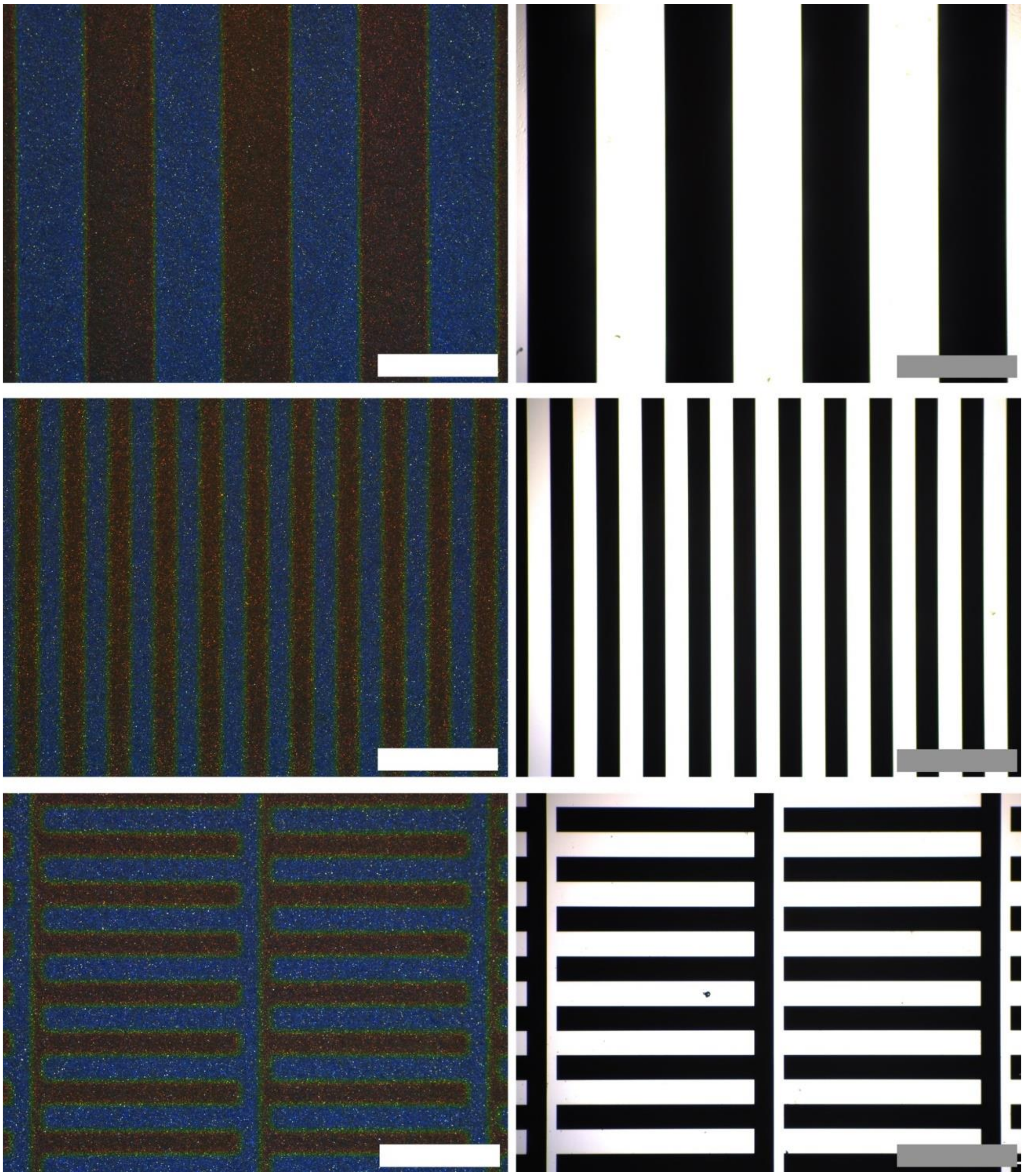

Fig. S8 Micrographs of CLCs imprinted with different patterns (left) and the respective photomasks (right) employed in the photoisomerization process. The images were acquired on a polarized optical microscope (POM) operating in reflectance mode. Scale bars are 500 µm. Using the photoisomerization technique it is possible to achieve different colors in the same cholesteric layer with remarkable resolution. The details in the boundaries of the patterns are related to the UV light scattering caused by the photomask during photoisomerization. The boundary defects are in the order of a few microns and imperceptible to the naked eyes.

Tab. S1 Overview on strategies for preparing multicolor mechanochromic films.

**Material/reference**	**Patterning strategy**	**Approx. resolution** ***(order of magnitude)***	**Full-colored patterns**
Cholesteric liquid crystal elastomer *(this work)*	Photoisomerizable chiral compound	µm	Yes
Commercially available photosensitive elastomer [3]	Adapted Lippman photography	mm	Yes
Cholesteric liquid crystal elastomer [4]	Temperature + multiple crosslinking steps	mm	No
Cholesteric liquid crystal elastomer [5]	Temperature + multiple crosslinking steps	mm	No
Nanoparticle-based photonic crystal in an elastomer matrix [6]	Control of the crosslinking degree	mm	No
Cholesteric liquid crystal elastomer [7]	Direct ink writing	cm	Yes
Cholesteric liquid crystal elastomer [8]	Control of the elastic modulus	cm	Yes
Cholesteric liquid crystal elastomer [2]	Control of the crosslinking degree	cm	No
Cholesteric liquid crystal elastomer [9]	Temperature + multiple crosslinking steps	cm	No
Nanoparticle-based photonic crystal in an elastomer matrix [10]	Crosslinking the pattern and washing the background	cm	No

REFERENCES

[1] J. Lub, W. P. M. Nijssen, R. T. Wegh, J. P. A. Vogels, A. Ferrer, *Adv Funct Mater* **2005**, *15*, 1961–1972.

[2] L. D. C. de Castro, T. A. P. Engels, O. N. Oliveira, A. P. H. J. Schenning, *ACS Appl Mater Interfaces* **2024**, *16*, 14144–14151.

[3] B. H. Miller, H. Liu, M. Kolle, *Nat Mater* **2022**, DOI 10.1038/s41563-022-01318-x.

[4] X. Shi, Z. Deng, P. Zhang, Y. Wang, G. Zhou, L. T. de Haan, *Adv Funct Mater* **2021**, *31*, DOI 10.1002/adfm.202104641.

[5] P. Zhang, X. Shi, A. P. H. J. Schenning, G. Zhou, L. T. de Haan, *Adv Mater Interfaces* **2020**, *7*, DOI 10.1002/admi.201901878.

[6] S. Ye, Q. Fu, J. Ge, *Adv Funct Mater* **2014**, *24*, 6430–6438.

[7] J. Choi, Y. Choi, J. H. Lee, M. C. Kim, S. Park, K. Hyun, K. M. Lee, T. H. Yoon, S. kyun Ahn, *Adv Funct Mater* **2023**, DOI 10.1002/adfm.202310658.

[8] S. Nam, D. Wang, C. Kwon, S. H. Han, S. S. Choi, *Adv Mater* **2023**, DOI 10.1002/adma.202302456.

[9] L. Lu, X. Chen, W. Liu, H. Li, Y. Li, Y. Yang, *Liq Cryst* **2023**, DOI 10.1080/02678292.2023.2200266.

[10] G. H. Lee, S. H. Han, J. Bin Kim, J. H. Kim, J. M. Lee, S.-H. Kim, *Chem Mater* **2019**, *31*, 8154–8162.
